# Neuropsychiatric Symptoms in Parkinson's Disease with Mild Cognitive Impairment and Dementia

**DOI:** 10.1155/2012/308097

**Published:** 2012-08-30

**Authors:** Iracema Leroi, Hiranmayi Pantula, Kathryn McDonald, Vijay Harbishettar

**Affiliations:** ^1^Institute of Brain, Behavior and Mental Health, School of Community-Based Medicine, University of Manchester, Jean McFarlane Building, Oxford Road, Manchester M13 9PL, UK; ^2^Manchester Mental Health and Social Care Trust, Manchester M21 9UN, UK; ^3^Lancashire Care NHS Foundation Trust, Lytham Hospital, Warton Street, Lytham FY8 5EE, UK; ^4^University of Manchester, Oxford Road, Manchester M13 9PL, UK; ^5^Salford Royal NHS Foundation Trust, Salford M6 8HD, UK; ^6^Academic Clinical Psychiatry, University of Sheffield, Sheffield S5 7JT, UK

## Abstract

Neuropsychiatric symptoms commonly complicate Parkinson's disease (PD), however the presence of such symptoms in mild cognitive impairment (PD-MCI) specifically has not yet been well described. The objective of this study was to examine and compare the prevalence and profile of neuropsychiatric symptoms in patients with PD-MCI (*n* = 48) to those with PD and no cognitive impairment (PD-NC, *n* = 54) and to those with dementia in PD (PDD, *n* = 25). PD-MCI and PDD were defined using specific consensus criteria, and neuropsychiatric symptoms were assessed with the 12-item Neuropsychiatric Inventory (NPI). Self-rated apathy, depression, and anxiety rating scales were also administered. Over 79% of all participants reported at least one neuropsychiatric symptom in the past month. The proportion in each group who had total NPI scores of ≥4 (“clinically significant”) was as follows: PD-NC, 64.8%; PD-MCI, 62%; PDD 76%. Apathy was reported in almost 50% of those with PD-MCI and PDD, and it was an important neuropsychiatric symptom differentiating PD-MCI from PD-NC. Psychosis (hallucinations and delusions) increased from 12.9% in PD-NC group; 16.7% in PD-MCI group; and 48% in PDD group. Identifying neuropsychiatric symptoms in PD-MCI may have implications for ascertaining conversion to dementia in PD.

## 1. Introduction

In Parkinson's disease (PD), cognitive impairment and the development of dementia (PDD) are increasingly being considered part of the disease course. Mild cognitive impairment in PD (PD-MCI) occurs in about 25% of patients and may predict conversion to PDD [1, 2]. Formal diagnostic criteria for PD-MCI have recently been proposed by the Movement Disorder Society (MDS) Task Force [3]. Risk factors for the development of PD-MCI include older age at disease onset, male gender, depression, severity of motor symptoms, and advanced disease stage [4].

According to the MDS Task Force proposal, PD-MCI is a syndrome defined by three sets of criteria: clinical, cognitive and functional. The proposed cognitive criteria comprise two levels of assessment. Level I involves an abbreviated assessment using a global scale of cognition or limited neuropsychological test batteries for a diagnosis of “possible PD-MCI.” Level II involves more extensive neuropsychological testing using tests in five domains, with impairment on at least two tests in one or more domains for a diagnosis of PD-MCI subtypes. The domains are attention and working memory, executive dysfunction, language, memory, and visuospatial function. PD-MCI predominantly affects the memory, visual-spatial, and attention/executive domains, with the most common subtype being “non-amnestic single domain” MCI [3].

Since PD-MCI is a newly defined entity, extensive studies examining the clinical features, associated factors, prognosis, and response to interventions have not yet been undertaken. In particular, the psychiatric and behavioural symptoms of PD-MCI defined in this way are not yet well understood. The MDS Task Force report specifically points out that although psychiatric complications such as psychosis or apathy have been associated with PDD, “there is insufficient evidence to recommend that the presence of these symptoms strongly supports a diagnosis of PD-MCI.” Greater understanding of PD-MCI is critical in order to determine the impact of this entity on patients and caregivers and whether or not these non-cognitive aspects of PD-MCI are risk factors for conversion to PDD.

 Neuropsychiatric symptoms form part of the constellation of non-motor symptoms in PD which has a significant impact on the quality of life of PD patients, as well as caregiver burden and distress [5–8]. The most common neuropsychiatric symptoms in PD, regardless of cognitive status, are depression and hallucinations [5]. However, the frequency of these and other neuropsychiatric symptoms in PD patients with MCI is not known. A population-based study of 824 people *without* PD revealed that the prevalence of these symptoms in those with MCI is as high as 43%, with 29% having “clinically significant” symptoms [9]. Neuropsychiatric symptoms are more prevalent in older people who meet criteria for MCI compared to those who have cognitive impairment that have not yet met MCI criteria [10]. 

Compared with PD patients without dementia, those with PDD have a much greater prevalence (up to 89%) of at least one neuropsychiatric symptom, and 77% have two or more neuropsychiatric symptoms [11]. In a study that examined clusters of neuropsychiatric symptoms and cognitive status in PD, it was found that PDD was most commonly represented in the cluster characterised by hallucinations (79.3% had PDD), mixed neuropsychiatric symptoms (57.1% had PDD), and mild depression (31% had PDD) [12]. The lowest PDD representation within a cluster was in the sleep disturbances group (7.1% had PDD). Patients in the hallucination cluster also tended to have longer disease duration, more severe motor symptoms, and older age. Another cluster analysis, this time in PD patients without dementia, revealed clustering into five sub-groups: apathy, psychosis, depression, anxiety, and “low total neuropsychiatric symptoms.” Patients with “low total neuropsychiatric symptoms” had more preserved cognitive function [11].

It is important to assess the prevalence, profile, and magnitude of neuropsychiatric symptoms in PD-MCI since it is likely that the majority of PD-MCI sufferers are still functionally unimpaired, in active employment and may be suffering under an added burden of behavioural symptoms. Furthermore, neuropsychiatric symptoms may have prognostic implications and may be a risk factor for conversion to PDD amongst those who fall within the PD-MCI group. The aim of this present study was to (1) compare the frequency, magnitude and profile of neuropsychiatric symptoms in PD with intact cognition, PD-MCI, and PDD and (2) to explore the relationship of neuropsychiatric symptoms in these groups with their motor and cognitive profiles. We hypothesised that there would be an increase in the frequency and magnitude of neuropsychiatric symptoms as cognition declined across the groups in a pattern of PD without cognitive impairment (PD-NC) < PD-MCI < PDD. Furthermore, we hypothesised that the core psychiatric syndromes of apathy and psychosis would be more frequent and of greater magnitude as cognitive impairment developed. 

## 2. Methods

This study was approved by a regional ethics committee, and all participants and their informants gave informed consent. For participants with cognitive impairment in whom the capacity to consent may have been in doubt, caregivers were asked to sign an additional “assent” form. 

### 2.1. Participants and Classification of Cognitive Groups

Participants (*n* = 127) with idiopathic PD, diagnosed according to UK Brain Bank criteria, were consecutively recruited from community-based PD clinics in the North West of England as part of two clinical research protocols [13]. Of these, the data for the participants with a diagnosis of PDD (*n* = 25) were part of a randomised-controlled clinical trial of memantine, and data for the current study were taken from the baseline assessments [14]. The participants without dementia (PD-NC, *n* = 54; PD-MCI, *n* = 48) were recruited as part of the current descriptive study. In all cases, the screening evaluation, involving a neurologic and mental state exam, cognitive screen, and informant interview for collateral information, was undertaken to determine whether criteria for probable PDD were met [15]. All assessments were done during the “on” motor state. Participants' medication for the motor aspects of PD remained unchanged for at least four weeks prior to and during the study, and no participants were taking anticholinergic medications at the time of the assessment. 

The criteria for PDD were according to the MDS Task Force criteria for PDD and operationalised according to the diagnostic algorithm outlined by Dubois et al. (2007) [15, 16]. Briefly, this involved the following: (1) onset of cognitive impairment after the onset of motor symptoms; (2) decreased global cognitive efficiency as evidenced by a Mini-Mental State Exam [17] (MMSE; score of <26); (3) functional impairment due to cognitive deficits, determined by caregiver reports; (4) deficits in more than one cognitive domain (attention, executive function, visuospatial functioning, memory, and language). 

 The syndrome of PD-MCI (*n* = 48) was identified in those who did not meet PDD criteria, had a MMSE score of ≥26, and who met the proposed inclusion and exclusion criteria for this category according to the MDS Task Force [3]. Briefly, this involved (1) gradual cognitive decline reported by the patient, clinician, or caregiver; (2) cognitive deficits on at least two tests of a formal neuropsychological battery with deficits defined as at least 1.5 SD more impaired than the mean scores for an age- and gender-matched healthy control group; (3) cognitive deficits not severe enough to significantly interfere with functional ability or activities of daily living as determined by caregiver or patient report. The specific neuropsychological battery chosen was a short, pragmatic battery that was tolerated by the participant group in the context of a wider study involving further assessments. The test battery comprised four of the five MDS Task Force recommended domains (Table 1), including three tests of attention and working memory, three tests of executive dysfunction, one test of memory, and one test of visuospatial function. This is consistent with Level 1 of the PD-MCI criteria which precluded the definition of specific PD-MCI subtypes.

The remaining participants were classified as PD-NC (*n* = 54). Premorbid intellect was assessed using the National Adult Reading Test (NART) and no significant differences were found between groups [23]. Each participant had a caregiver or informant who knew them well, had contact at least once a week, and could provide information on the participant's behaviour. Finally, in order to establish the norms on the cognitive battery, from which to derive the PD-MCI criteria, a healthy control group (*n* = 33) was recruited from non-caregiver acquaintances of the PD participants. This group was age-, culture-, education- and gender-matched to those with PD. All participants in this group were free of significant medical problems. They were assessed using the same battery as outlined in Table 1. 

### 2.2. Assessment

The Unified Parkinson's Disease Rating Scale part III was used to assess motor severity, and stage of disease was categorised according to the Hoehn-Yahr scale [24, 25]. In the two groups without dementia, levodopa daily equivalent dose (LEDD) was calculated according to a recommended formula for total dopaminergic replacement as well as for dopamine agonists only [26]. 

Neuropsychiatric symptoms were assessed using the 12-item Neuropsychiatric Inventory (NPI) according to published procedures [27]. The NPI is a validated informant-rated scale which assesses 12 domains of behavioural disturbance including delusions, hallucinations, agitation/aggression, dysphoria/depression, anxiety, apathy, euphoria, disinhibition, irritability/lability, aberrant motor behaviour, appetite, and sleep disturbances. Each domain is rated on presence and magnitude of symptoms (frequency × severity). The maximum score per domain is 12, with clinically significant symptoms for a given domain occurring at (frequency × severity) scores ≥4. Total NPI scores range from 0 to 144, with higher scores indicating greater behavioural disturbance. The NPI has been extensively used in PD and has been shown to be valid in PD populations both with and without dementia [5, 11].

Since caregiver ratings may be influenced by such factors as stress, depression, and burden in the caregiver, we included two self-rated scales for the assessment of psychopathology in the PD-NC and PD-MCI groups. These scales were the Hospital Anxiety and Depression Rating Scale (HADS) and the Apathy Scale [28, 29]. They were not administered to the PDD group because both scales depend upon the ability of the participant to be able to report on their own symptoms and are therefore less valid once dementia emerges [30, 31]. Since apathy was hypothesised as being a core psychiatric syndrome that might differentiate PD-NC from PD-MCI, it was further assessed in the two groups without dementia using the Apathy Inventory (AI). The AI, which is an informant-rated scale, is scored in a similar manner to the NPI (i.e., frequency × severity) and assesses three dissociable dimensions of apathy: emotional blunting, lack of initiative, and lack of interest [32]. Data for the AI were not available for the PDD group. 

### 2.3. Statistical Analysis

Analysis was performed using SPSS version 16 [33]. Initial univariate analysis comparing proportions among the three groups was undertaken using the chi-square (*χ* ^2^) test, and comparison of group differences was undertaken using either ANOVA (with *post hoc* Bonferroni two-group comparisons) or the Kruskall-Wallis (with *post hoc* Mann-Whitney *U* comparison) tests, depending on the distribution of the data. ANCOVA was used to control for confounding variables, where appropriate. Bivariate correlations (Pearson or Spearman) were also performed to explore associations of neuropsychiatric symptoms with demographic, motor, and cognitive variables. 

## 3. Results

### 3.1. Sample Characteristics

The majority of participants in each group was male, and the mean (SD) age across the entire group was 65.40 (11.16) years. The mean (SD) education level was 12.81 (2.80) years of formal education. The mean (SD) duration of PD was 93.77 (64.10) months, and mean MMSE was 26.72 (4.71), range (10–30). The median Hoehn and Yahr stage was 2.50 (interquartile range 2-3). 

Demographic and clinical characteristics of the three groups are outlined in Table 2. The mean age at time of assessment and at onset of motor symptoms differed significantly across the three groups in the order of PD-NC < PD-MCI < PDD. However, duration of disease did not differ among the groups and all participants were between Hoehn-Yahr stages 2-3. Motor severity, as measured by the UPDRS part III, was worse in the PD-MCI group compared to the other two groups, which did not differ from each other. The PDD group had significantly fewer complications of therapy, as measured by the UPDRS part IV, compared to both groups without dementia. None of the PDD group participants was in active employment at the time of the study. Dopaminergic replacement load (calculated as levodopa equivalent daily dose, LEDD) did not significantly differ between the two groups without dementia. However, LEDD for dopamine agonists only was significantly lower in the PD-MCI group compared to the PD-NC group. 

Cognitive measures for the two PD groups without dementia as well as the healthy control group are shown in Table 3. For all cognitive tests across the four domains, the PD-MCI scores were significantly worse than both the PD-NC group and the healthy control group, which did not significantly differ from each other. The proportion of those in the PD-MCI group who were impaired in each of the specific domains was as follows (in ascending order): memory, 29%; attention and working memory, 37%; visuospatial function, 58%; executive function (“FAS” test and computerised Wisconsin Card Sorting Test), 60.4%. 

### 3.2. Comparison of Neuropsychiatric Symptoms in the Three PD Groups

#### 3.2.1. Informant-Rated

At least one neuropsychiatric symptom on the NPI was reported by 97 (77.9%) of the 127 participants. The most common neuropsychiatric symptoms were sleep disturbances (53.1%), anxiety (40.6%), dysphoria/depression (38.3%), apathy (35.2%), irritability/lability (25%), hallucinations (16.4%), and agitation/aggression (12.5%). The remaining NPI domains were present in less than 10% of the sample. The most commonly reported “clinically significant” neuropsychiatric symptoms (NPI domain score ≥ 4) were sleep disturbances (37.5%), apathy (25.8%), anxiety (10.9%), and dysphoria/depression (9.4%). The remaining NPI domains were “clinically significant” in less than 10% of the sample. The mean (SD) total NPI score across the entire sample was 11.61 (12.44).

Table 4 outlines the mean neuropsychiatric symptom scores (frequency × severity) in each NPI domain for the three groups, which were not significantly different. For the individual NPI behavioural domains, the key differences between groups were driven by the PDD group having significantly worse mean scores compared to both the PD-NC and PD-MCI groups in the domains of delusions, aberrant motor behavior, and disorders of appetite. Hallucinations were significantly worse in the PDD group compared to the PD-NC group only. The only significant difference in any of the NPI behavioural domains between the PD-NC and PD-MCI groups was apathy. This difference was also reflected in the differences in the more detailed caregiver-rated Apathy Inventory (AI). On the AI, two of the three subdomains (“lack of interest” and “lack of initiative”) were significantly greater in the PD-MCI group compared to the PD-NC group and the subdomain of “emotional blunting” reached a trend towards significance.

In both the PD-NC and the PD-MCI groups, the domains with the greatest magnitude (frequency × severity) were sleep, apathy, and anxiety. In the PDD group, the highest magnitude NPI domains were apathy followed by sleep, then irritability, depression, and anxiety. In the two groups without dementia, the majority of domains had mean magnitude scores <1.0 (not clinically significant), whereas in the PDD group, only three of the 12 domains (disinhibition, elation, agitation/aggression) had mean scores <1.0. 

In the PD-NC group, 39 (72.2%) participants reported at least one neuropsychiatric symptom compared to 38 (79.2%) in the PD-MCI and 24 (96%) in the PDD groups (*χ* ^2^ = 6.32; *P* = 0.04; PD-NC *versus* PDD, *P* = 0.01). Those with total NPI scores of ≥4 (“clinically significant”) were as follows: PD-NC, 64.8%; PD-MCI, 62%; PDD, 76% (*χ* ^2^=4.48; *P* = 0.10).  Table 5 shows the proportion of patients in each of the three groups who endorsed “any” or “clinically significant” (NPI ≥ 4) symptoms in each of the NPI domains. The most commonly reported psychiatric symptoms (reported in over 20% of participants and excluding sleep and appetite) in each of the three groups was as follows: (1) PD-NC, anxiety, dysphoria/depression and irritability/lability; (2) PD-MCI, apathy, anxiety and dysphoria/depression; (3) PDD, dysphoria/depression, apathy, irritability/lability, anxiety, agitation/aggression, hallucinations, delusions and aberrant motor behaviour. The only neuropsychiatric symptom which differed significantly in frequency between PD-MCI and PD-NC was apathy, which was reported almost as frequently in PDD as in PD-MCI (52% and 48% resp.). Of all those reporting “any” apathy (entire PD sample), 60% also reported any “any” depression. However, once clinically significant apathy only was considered (NPI apathy ≤ 4), the proportion of those also reporting clinically significant depression (NPI dysphoria/depression ≤ 4) as well decreased to 11.8%. Sleep problems were reported in >40% in all three groups with the two groups without dementia endorsing this domain most frequently (55% in PD-NC and 58% in PD-MCI). In contrast, appetite problems were reported in <10% in the two groups without dementia but were endorsed by 20% (16% were “clinically significant) of the PDD group. 

In contrast to the PD-MCI group, those with PDD endorsed neuropsychiatric symptoms in several domains significantly more frequently compared to the two groups without dementia. These domains were delusions, agitation/aggression, dysphoria/depression, irritability/lability, and aberrant motor behaviour. The proportions reporting apathy and hallucinations were significantly different between the PDD and PD-NC groups only. It was notable that psychosis (the presence of any hallucinations, delusions, or both) increased markedly with the extent of cognitive impairment: 12.9% in the PD-NC group, 16.7% in the PD-MCI group, and 48% in the PDD group (*χ* ^2^ = 14.26; *P* = 0.001). Finally, over 30% of each of the two groups without dementia endorsed the domains of anxiety and depression. For the PDD group, these figures increased to 48% and 56% for the two domains, respectively. 

#### 3.2.2. Self-Rated

As shown in Table 4, self-rated anxiety (HADS-A) did not differ significantly among the two PD groups without dementia and the healthy control group. However, in the PD-MCI group, both self-rated depression (HADS-D) and self-rated apathy (AS) were significantly worse compared to both the PD-NC and the healthy control groups. Furthermore, the PD-NC group was also significantly more depressed than the healthy control group. Since the PD-MCI group was significantly older, had worse motor function and had a lower dopamine agonist load compared to the PD-NC group, ANCOVA was performed for the self-rated depression and apathy scores with these variables as covariates. This revealed that the initial differences seen between the two groups remained significant for both depression (*F* = 4.81; *P* = 0.001) and apathy (*F* = 9.33;  *P* < 0.001). 

### 3.3. Correlation of Neuropsychiatric Symptoms with Other Key Variables

In the entire study group, significant correlations were seen between neuropsychiatric symptoms (represented by NPI total) and the following variables: duration of disease (*ρ* = 0.20; *P* = 0.03), Hoehn-Yahr stage (*ρ* = 0.23; *P* = 0.01) and MMSE score (*ρ* = −0.20; *P* = 0.02). Significant correlations were not seen between this variable and age, LEDD, age of disease onset, and motor severity (UPDRS motor and complications of therapy subscores). As shown in Table 6, psychosis (NPI delusions or hallucinations) also correlated with disease staging, MMSE, duration of disease, and age. Both self-rated apathy (in those without dementia) and informant-rated apathy (in the entire study sample) had several significant correlations, including markers of advanced disease (disease stage, MMSE, and motor severity), as well as dopamine agonist load (LEDD-dopamine agonist only), age, and age of disease onset (see Table 6). Finally, as is also shown in Table 6, in the participants without dementia, self-rated apathy was shown to have significant correlations with all the cognitive measures, whereas self-rated depression correlated with working memory (digit *n*-back) and verbal fluency (FAS) only. In contrast, self-rated anxiety did not correlate significantly with any of the cognitive measures.

## 4. Discussion

To our knowledge this is the first study to specifically examine neuropsychiatric symptoms in MCI related to PD identified using the new specific MDS Task Force criteria. This is a group of PD patients who have cognitive impairment but functional impairment not severe enough to warrant a diagnosis of dementia. They may, nonetheless, have non-motor symptoms that impact significantly on quality of life and other aspects of functioning. An understanding of the clinical correlates, particularly the neuropsychiatric correlates, of PD-MCI is crucial because this clinical entity has prognostic implications and may predict conversion to dementia. In addition, by examining non-motor manifestations such as neuropsychiatric symptoms, it may be possible to intervene and delay the conversion to dementia. In this study, we hypothesised that there would be an increase in the frequency and magnitude of neuropsychiatric symptoms as cognition declined across the groups from PD without cognitive impairment to PDD. Our findings only partially supported this presentation. Rather, aside from apathy and self-rated depression, the two groups without dementia appeared quite similar with regards to neuropsychiatric symptoms. In contrast, the PDD group was distinguished from both comparator groups by a greater proportion of neuropsychiatric symptoms (both presence of “any” symptom and “clinically significant” symptoms) across several domains as well as a higher magnitude and frequency of psychosis (delusions and hallucinations) and aberrant motor behaviour. Only apathy was as frequent in the PD-MCI as in the psychiatrically more vulnerable PDD group. 

The finding that the frequency and magnitude of neuropsychiatric symptoms were similar in the PD-NC and PD-MCI groups diverges from the pattern that has been described for neuropsychiatric symptoms in MCI in the general population. In this case, the prevalence of neuropsychiatric symptoms was midway between the prevalence in healthy control participants and in those with dementia [9]. These findings were felt to support the notion that MCI was a precursor to dementia, as may be the case in PD. According to evidence building from longitudinal studies in PD, MCI is likely to be a precursor to dementia. Based on the findings in our study, it may be possible to identify conversion from PD-MCI to PDD with the emergence of significant neuropsychiatric symptoms. The profile of symptoms in our PDD sample was similar to previously reported PDD samples however we did not examine “clusters” of symptoms but instead examined individual NPI domains. 

It is noteworthy that apathy was the key neuropsychiatric feature distinguishing the two groups without dementia, and it was evident on both informant- and self-rated scales. This suggests that the apathy syndrome is closely linked to cognitive impairment and may even be a harbinger of conversion to dementia, a finding that has previously been observed. For example, a recent longitudinal study of a PD cohort without dementia found that after a median period of 18 months, the proportion of those who converted to dementia was significantly higher in those with apathy [34]. Moreover, in those who did not develop dementia, cognitive decline was still greater in the apathy sufferers. In our study, the correlation between apathy and all the cognitive measures tested supports the notion of a very tight link between these factors. 

In PD populations without dementia, apathy has been associated with older age, older age of disease onset, psychiatric complications, greater global cognitive impairment, and lower dopamine agonist load as well as depression [35]. In our sample, apathy was also associated with all these factors as well as dopamine agonist load and motor severity, even though the UPDRS motor score in the PDD group was lower than in the PD-MCI group. The lower severity score may reflect the loss of tremor that has been associated with the onset of dementia in PDD [36]. Since age, age of onset and cognitive impairment all increased across the groups from PD-NC to PDD, the emergence of apathy might be accounted for by these factors rather than the presence of the “MCI” status *per se*. Nonetheless, the differences in apathy between the PD-NC and the PD-MCI group remained significant even after controlling for possible confounding factors such as age and motor severity. Finally, the difference in dopamine agonist load between the PD-NC and PD-MCI groups needs to be considered as a possible factor in the appearance of apathy in those with MCI. Dopamine and dopamine agonists may have a role in reward and motivation processing, and it is possible that with the emergence of cognitive impairment and more advanced disease in the PD-MCI group, dopamine agonists were prescribed more sparingly, which may have contributed to the emergence of apathy [37, 38]. In addition, identifying apathy in those with PD without dementia is important, due to the impact apathy has on level of disability and caregiver burden [5, 6, 8]. 

The increased level of self-rated depression in the PD-MCI group was also of considerable interest. It is possible that as cognitive changes start to appear, particularly the point of PD-MCI, symptoms of depression are seen as well. This was supported by the positive correlations between depression ratings and measures of executive dysfunction (working memory and verbal fluency). An overlap between depression and apathy was also seen; however, as apathy severity increased, the proportion who also reported co-morbid depression decreased. This suggested the emergence of a “purer” form of apathy. The association of apathy with depression in PD is complex and there are studies which have shown a significant level of discrepancy between apathy and depression. For example, a longitudinal study of 65 patients with Alzheimer disease found that apathy and depression had different natural histories which are possible to discriminate [39]. On another note, the high rates of both anxiety and depression in the two groups without dementia should also be considered. This finding corroborates evidence from an Australian survey of patients with PD without dementia, where the occurrence of anxiety disorders was found to be 25% [40]. 

 Our study demonstrates that the profile of neuropsychiatric symptoms differed in those with PDD compared to those without dementia. In particular, a greater number of specific neuropsychiatric symptoms were reported by over a quarter of those with PDD compared to the other two groups. Moreover, the symptoms of psychosis (hallucinations and delusions) emerged as clinically significant and frequent. Psychosis was associated with markers of advanced disease (longer disease duration, later disease stage, lower MMSE) as well as increasing age. This pattern supports previous findings in which psychotic symptoms increased linearly with degree of cognitive impairment when comparing PD without cognitive impairment to PDD [41]. Both psychosis and significant cognitive impairment in PD have been associated with cholinergic deficits and may be improved with the use of cholinesterase inhibitors [42, 43].

Limitations to the current study were that the sample size was relatively small and that the participants were consecutively drawn from a convenience sample in community clinics, rather than using strict epidemiologic methods. Furthermore, we examined neuropsychiatric symptoms as single domains whereas studies using cluster analysis have demonstrated that groups of neuropsychiatric symptoms are linked. Further investigation of such clusters in PD-MCI might prove fruitful [12]. Another limitation was that the neuropsychiatric tool used in this study (the NPI) is informant-rated and may therefore be subject to bias due to caregiver factors such as distress, fatigue, and depression. In addition, the current methods did not enable us to specifically determine whether or not our findings in relation to the neuropsychiatric symptoms were due to cognitive state, rather than other factors such as stage of disease or differences in age. However, this is less likely since disease duration did not differ among groups. Finally, our neuropsychological battery was designed to be short and pragmatic, and the tests chosen in each domain, particularly the memory domain, were restricted and did not enable us to subtype the PD-MCI group. Nonetheless, the battery was still able to fulfil the MDS Task Force Level I criteria for PD-MCI and may reflect findings in a clinical setting where more extensive and time-consuming test batteries are not practicable or tolerated. 

In conclusion, this study found that neuropsychiatric symptoms are increasingly prevalent with increasing levels of cognitive impairment in PD, particularly as dementia emerges. Identifying PD-MCI as a clinical entity and estimating neuropsychiatric symptoms, particularly apathy, in this group can aid in understanding the risk for conversion to dementia. To our knowledge, this is the first study to specifically examine the prevalence and magnitude of such symptoms in a group of PD participants identified as PD-MCI. A deeper understanding of these symptoms may guard against a “hypercognitive” definition of PD-MCI. 

## Figures and Tables

**Table 1:** Pragmatic neuropsychological test battery and cognitive domains administered to the PD participants without dementia.

Cognitive domain	Neuropsychological test
Attention and working memory	Trail Making Test A and B [18] Serial 7's; Digit *n*-back [19]^i^
	“FAS” verbal fluency task [20]
Executive dysfunction	WCST^ii^ [21]
	Digit *n*-back
Memory	5-minute recall of 3 words
Visuospatial function	Intersecting pentagons

^i^The digit *n*-back evaluates working memory, which comprises both attentional and executive components of short-term memory. This task has been used in PD (e.g., [22]). ^ii^Computerised version of the Wisconsin Card Sorting Test.

**Table 2:** Demographics and clinical characteristics of PD participants by group: without cognitive impairment, mild cognitive impairment, and dementia.

	PD	PD		Statistic (*F* or *χ* ^2^); *P* value
	No cognitive	Mild cognitive	PD dementia	
	impairment	impairment	(PDD; *n* = 25)	
	(PD-NC; *n* = 54)	(PD-MCI; *n* = 48)		
	Mean (SD) or *n* (%)			

Demographics				
Age (years)	58.11 (9.87)	68.63 (8.39)	75.58 (7.47)	23.30; <0.001^a, b, c^
Male gender *n* (%)	38 (71.7)	34 (70.83)	13 (52.0)	3.05; 0.22
Married *n* (%)	40 (75.5)	40 (83.3)	14 (56.0)	0.65; 0.42
In active employment *n* (%)	14 (25.9)	3 (6.2)	0 (0)	136.38; <0.001

Disease characteristics				
Age of PD onset (years)	50.62 (9.81)	60.02 (11.56)	66.68 (10.95)	21.35; <0.001^a, b, c^
Duration of PD (months)	87.74 (47.57)	103.66 (79.84)	86.85 (60.94)	0.92; 0.40
UPDRS^i^ motor score	25.33 (12.36)	31.26 (10.61)	24.12 (9.89)	4.69; 0.01^a, b^
UPDRS complications of therapy	3.70 (3.25)	3.66 (3.37)	0.42 (1.25)	11.26; <0.001^b, c^
LEDD^ii^	759.90 (537.85)	852.48 (625.56)	Not obtained	0.63; 0.43
LEDD-DA^iii^	208.81 (174.85)	67.95 (110.99)	Not obtained	22.11; <0.001
Hoehn-Yahr staging	2.04 (0.65)	2.28 (0.65)	3.40 (0.80)	23.39; <0.001^b, c^
MMSE^iv^	29.30 (0.82)	27.64 (1.90)	19.36 (5.96)	102.74; <0.001^a, b, c^
Pre-morid IQ (NART^v^)	113.48 (9.84)	112.1 (11.77)	110.45 (7.98)	0.18; 0.84

^i^Unified Parkinson's Disease Rating Scale; ^ii^total daily dopaminergic load based on levodopa equivalents or “levodopa equivalent daily dose” (LEDD); ^iii^LEDD-DA: levodopa equivalent daily dose-dopamine agonist only; ^iv^Mini-Mental State Exam; ^v^National Adult Reading Test [28].

*Post hoc* bonferroni for two group comparison, *P* < 0.05: ^a^PD-NC *versus* PD-MCI; ^b^PD-MCI *versus* PDD; ^c^PD-NC *versus* PDD.

**Table 3:** Neuropsychological test scores for the PD groups without cognitive impairment and with mild cognitive impairment, and the healthy control group.

	PD	PD		Statistic (*F* or *χ* ^2^); *P* value
	No cognitive	Mild cognitive	Healthy control	
	impairment	impairment	(HC; *n* = 33)	
	(PD-NC; *n* = 54)	(PD-MC; *n* = 48)		
	Mean (SD)			

Attention and working memory				
Trails B-Trails A (reaction time in seconds)	41.55 (17.41)	136.68 (71.30)	41.27 (26.23)	85.32; <0.001
Serial 7's	4.65 (0.59)	3.78 (1.40)	5.09 (0.52)	20.65; <0.001
Digit *n*-back	17.76 (3.24)	13.48 (2.76)	19.68 (3.09)	43.89; <0.001

Executive function				
cWCST total^ii^	39.04 (7.41)	32.32 (9.47)	40.82 (8.63)	11.55; <0.001
FAS total	47.89 (12.41)	34.06 (10.40)	52.71 (12.09)	26.84; <0.001

Memory				
5-minute recall of three words	2.72 (0.49)	2.20 (1.03)	2.78 (0.78)	7.62; 0.001

Visuospatial function				
Intersecting pentagons	0.98 (0.14)	0.40 (0.49)	1.00 (0)	71.95; <0.001

^ii^cWCST: Computerised version of the Wisconsin Card Sorting Test.

**Table 4:** Mean domain scores (frequency × severity) of the neuropsychiatric inventory items, apathy ratings, and self-rated depression and anxiety.

Psychiatric measure	PD	PD		Statistic (*F* or *t*); *P* value
	No cognitive	Mild cognitive	PD dementia	
	impairment	impairment	(PDD; *n* = 25)	
	(PD-NC; *n* = 54)	(PD-MCI; *n* = 48)		
Informant-rated scales:	Mean (SD)			

Neuropsychiatric Inventory (NPI) total and domain subscores				
NPI total score	9.53 (13.03)	12.38 (12.55)	14.56 (10.50)	1.55; 0.22
Delusions	0.19 (0.99)	0.15 (0.88)	1.08 (1.93)	5.68; 0.004^b, c^
Hallucinations	0.21 (0.70)	0.40 (1.35)	1.00 (1.97)	3.17; 0.045^c^
Agitation/aggression	0.33 (1.25)	0.34 (1.78)	0.84 (1.67)	1.06; 0.35
Dysphoria/depression	0.92 (1.67)	1.02 (1.91)	1.52 (1.80)	0.99; 0.37
Anxiety	1.04 (1.41)	1.21 (2.07)	1.36 (2.81)	0.23; 0.79
Elation	0.25 (0.96)	0.26 (1.75)	0.12 (0.60)	0.11; 0.89
Apathy	1.01 (2.62)	3.79 (4.91)	2.8 (3.87)	6.43; 0.002^a^
Disinhibition	0.15 (0.87)	0.13 (0.88)	0.24 (0.72)	0.15; 0.86
Irritability/lability	0.71 (2.00)	0.70 (2.19)	1.52 (2.33)	1.44; 0.24
Aberrant motor behaviour	0.25 (1.67)	0.13 (0.88)	1.20 (2.53)	3.77; 0.03^b, c^
Sleep	3.29 (3.75)	3.91 (4.08)	2.16 (3.39)	1.73; 0.18
Appetite	0.27 (1.03)	0.06 (0.44)	1.28 (3.05)	5.40; 0.006^b, c^

Apathy Inventory (AI)				
AI^vi^ total score	3.57 (7.61)	8.67 (11.77)	NA	6.05; 0.02
AI, emotional blunting	0.91 (2.11)	2.18 (4.03)	NA	3.54; 0.06
AI, lack of initiative	1.24 (3.01)	3.24 (4.50)	NA	6.28; 0.02
AI, lack of interest	1.41 (3.07)	3.24 (4.31)	NA	5.48; 0.02

	PD	PD		Statistic (*F*; *P* value)
	No cognitive	Mild cognitive	Healthy controls	
	impairment	impairment	(HC; *n* = 33)	
	(PD-NC; *n* = 54)	(PD-MCI; *n* = 48)		

Self-rated scales:	Mean (SD)			

HADS^vii^-anxiety subscore	6.15 (4.61)	6.24 (4.10)	4.50 (2.74)	2.10; 0.27
HADS-depression subscore	4.92 (3.67)	7.12 (3.50)	2.78 (2.59)	12.56; <0.001^a, d, e^
Apathy Scale	7.94 (8.54)	17.62 (11.92)	9.87 (5.09)	10.17; <0.001^a, e^

^vi^Apathy Inventory (AI); ^vii^Hospital Anxiety and Depression Scale.

*Post  hoc* bonferroni for two group comparison, *P* < 0.05: ^a^PD-NC *versus* PD-MCI; ^b^PD-MCI *versus* PDD; ^c^PD-NC *versus* PDD; ^d^PD-NC *versus* HC; ^e^PD-MCI *versus* HC.

**Table 5:** Proportion of Neuropsychiatric Inventory (NPI) domains endorsed by the three PD groups.

	PD		PD					
	No cognitive		Mild cognitive		PD dementia			
	impairment		impairment		(PDD; *n* = 25)		Statistic (*χ* ^2^; *P*-value)	
	(PD-NC; *n* = 54)		(PD-MCI; *n* = 48)					
	%							

NPI sub-scores	Any*	≥4**	Any	≥4	Any	≥4	Any	≥4

Delusions	3.7	3.7	4	2	28.0	12.0	28.40; <0.001^b, c^	10.08; 0.07
Hallucinations	11.1	1.9	14.0	4.0	32.0	12.0	37.21; <0.001^c^	18.2; 0.003^c^
Agitation/aggression	7.4	3.7	6.0	2	36.0	8.0	32.47; <0.001^b, c^	6.38; 0.23
Dysphoria/depression	33.3	9.3	36.0	6.0	56.0	20.0	48.92; <0.001^b, c^	15.07; 0.01^a^
Anxiety	42.6	11.1	36.0	12.0	48.0	12.0	43.62; <0.001^b, c^	9.19; 0.10
Euphoria	7.4	3.7	2.0	2.0	4.0	0.0	8.84; 0.12	8.53; 0.13
Apathy	16.7	11.1	48	38	52.0	36.0	50.99; <0.001^a, c^	37.43; <001^a, c^
Disinhibition	3.7	1.9	2	2	12.0	0.0	9.10; 0.11	4.79; 0.44
Irritability/lability	22.2	9.3	14.0	4.0	52.0	16.0	44.65; < 0.001^b, c^	11.75; 0.04^c^
Aberrant motor behaviours	5.6	3.7	0	0	24.0	16.0	23.18; <0.001^b, c^	15.49; 0.01^c^
Sleep	55.6	37.0	58.0	42.0	40.0	32.0	44.04; <0.001	52.93; <0.001^b^
Appetite	7.4	1.9	2.0	0	20.0	16.0	15.85; 0.003^c^	12.9; 0.01^c^

*Indicates any Neuropsychiatric Inventory (NPI) symptoms >0; **indicates NPI score of ≥4.

*Post  hoc* bonferroni for two group comparison, *P* < 0.05: ^a^PD-NC *versus* PD-MCI; ^b^PD-MCI *versus* PDD; ^c^PD-NC *versus* PDD.

**Table 6:** Correlations between behavioural scores and key demographic, disease, and cognitive variables.

	Self-rated	Informant-rated	
	(participants without dementia; *n* = 102)	(all participants; *n* = 127)	
	Apathy Scale	NPI^viii^-apathy	NPI-psychosis
			(hallucinations or delusions)
Demographic and disease variables	*ρ*; *P* value		

Age	0.39; 0.001	0.31; 0.001	0.19; 0.04
LEDD^ii^	0.01; 0.92	0.07; 0.52	0.07; 0.49
LEDD-DA^iii^	−0.38; <0.001	−0.33; 0.001	0.07; 0.47
Age of onset	0.30; 0.003	−0.21; 0.02	0.05; 0.61
Duration of illness	0.01; 0.89	0.13; 0.15	0.25; 0.006
Hoehn-Yahr	0.33; 0.001	0.25; 0.006	0.21; 0.02
UPDRS^i^ motor	0.33; 0.001	0.22; 0.02	0.02; 0.83
UPDRS complication	0.28; 0.93	−0.12; 0.19	−0.08; 0.39
MMSE^iv^	−0.41; <0.001	−0.21; 0.02	0.27; 0.003

	Self-rated scales in the participants without dementia (*n* = 102)		
	Apathy Scale	HADS^vi^-depression	HADS -anxiety

Cognitive measures	*ρ*; *P* value		

Trails B-Trails A (reaction time in seconds)	0.23; 0.03	−0.19; 0.07	−0.12; 0.30
Serial 7's	−0.27; 0.007	−0.08; 0.40	0.01; 0.88
Digit *n*-back	−0.29; 0.007	−0.23; 0.03	0.08; 0.45
cWCST^x^	−0.27; 0.01	−0.16; 0.12	0.08; 0.42
FAS^ix^ total	−0.23; 0.03	−0.20; 0.04	0.18; 0.08
5-min recall of three words	−0.21; 0.04	−0.14; 0.18	0.00; 1.00
Intersecting pentagons	−0.25; 0.02	−0.12; 0.20	0.07; 0.37

^i^Unified Parkinson's Disease Rating Scale; ^x^computerised version of the Wisconsin Card Sorting Test; ^ii^total daily dopaminergic load based on levodopa equivalents or “levodopa equivalent daily dose”; ^iii^levodopa equivalent daily dose-dopamine agonist only; ^iv^Mini-Mental State Exam; ^vi^HADS: Hospital Anxiety and Depression Rating Scale; ^viii^Neuropsychiatric Inventory; ^ix^verbal fluency FAS test.
